# Reduction of Dislocation of Large Joints, by Power Derived from Twisted Rope

**Published:** 1845-07

**Authors:** 


*Reduction of Dislocation of Large Joints by Power derived from Twisted Rope.*—Dr. Gilbert, Prof. of Surgery in Pennsylvania College, Phila., suggests a method of reducing dislocations of the large joints, which seems to combine the advantages of the pulleys, with greater simplicity, and with the important recommendation that the appliances are always at hand. He attributes to Dr. Fahnestock, of Pittsburg, the credit of first using it.

He describes the mode of application as follows: "Place the patient and adjust the extending and counter, extending bands as for the pulleys; then procure an ordinary bed cord, or wash line, tie the ends together, and again double it upon itself; then pass it through the extending tapes or towels, doubling the whole once more, and fasten the distal end, consisting of four loops of rope, to a window sill, door sill, or staple, so that the ropes are drawn moderately tight; finally, pass a stick through the centre of the doubled rope, dividing the strands equally by it; then, by revolving the stick as au axis or double lever, the power is produced precisely as it should be in such cases, viz:—slowly, steadily, and continuously." Its application is illustrated by a cut in the *Am. Jour. of Med. Sci.* No. for April ult. from which the above is taken. We commend this suggestion especially to surgeons in the country. It strikes us that it must prove an excellent substitute for pulleys, and is infinitely better than the clumsy and objectionable contrivances frequently employed under circumstances where recourse cannot readily be had to pulleys.—*Buff. Med. Jour.*

